# Correction to “Factors influencing the effectiveness of case management interventions for suicide attempters in a psychiatric hospital”

**DOI:** 10.1002/pcn5.70202

**Published:** 2025-09-07

Saito M, Shiratori Y, Yaguchi C, Yamada N, Ogawa T, Karashima M, Mizuhiki T, Hori T, Tachikawa H. Factors influencing the effectiveness of case management interventions for suicide attempters in a psychiatric hospital. PCN Rep. 2025 Aug 3;4(3):e70173.

In the published article, a minor typographical error was identified in Figure 2. The *y*‐axis label currently reads “Comulative” which is incorrect. The correct spelling should be “Cumulative.”

The corrected Figure 2 is provided below.


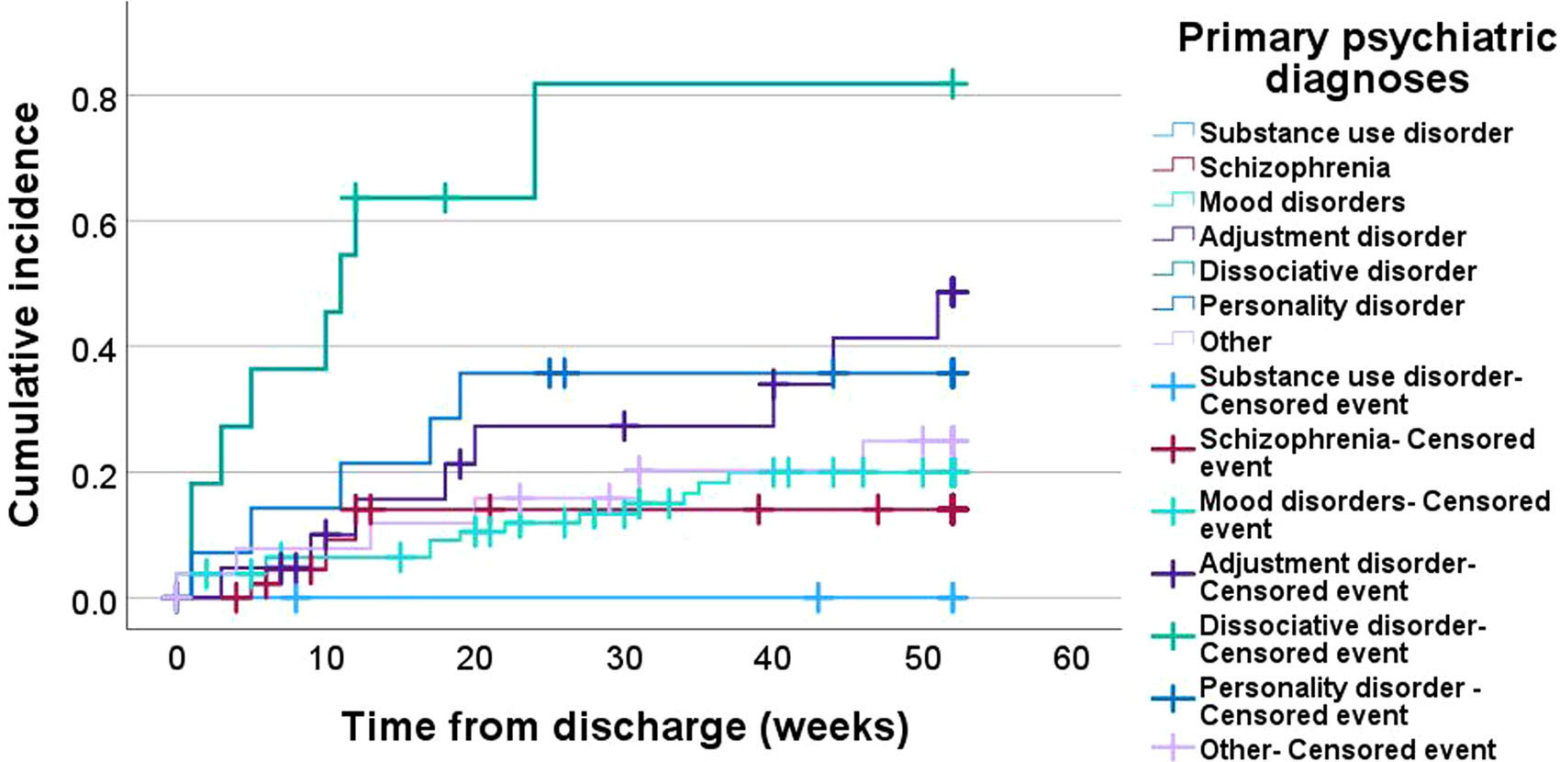


We apologize for this oversight.

